# Trends in benzodiazepine anxiolytics and z-hypnotics use among French drivers involved in road traffic crashes from 2005 to 2015: a responsibility case-control study

**DOI:** 10.1186/s40621-019-0209-8

**Published:** 2019-07-01

**Authors:** Ludivine Orriols, Gwladys Nadia Gbaguidi, Benjamin Contrand, Blandine Gadegbeku, Emmanuel Lagarde

**Affiliations:** 1 Univ. Bordeaux, ISPED, Centre INSERM U897-Epidemiologie-Biostatistique, F-33000 Bordeaux, France; 2 INSERM, IETO team, ISPED, Centre INSERM U1219-Epidemiologie-Biostatistique, F-33000 Bordeaux, France; 3 Université de Lyon, F-69000 Lyon, France; 4 IFSTTAR, UMR T 9405, UMRESTTE, F-69500 Bron, France; 5 Université Lyon 1, UMRESTTE, F-69000 Lyon, France

**Keywords:** Benzodiazepines, Z-hypnotics, Road traffic crashes, Pictogram

## Abstract

**Background:**

In France, benzodiazepine anxiolytics and z-hypnotics (zolpidem and zopiclone) account for the largest share of road traffic crash risk attributable to exposure to prescription drugs. The aim of this study was to monitor the evolution of the use of these prescription drugs and their association with crash risk over a period that began before the implementation of a color-graded pictogram system printed on prescription drug boxes.

**Methods:**

Data from three French national databases were extracted and linked: the national health care insurance database, police reports, and the national police database of injurious crashes. Drivers involved in an injurious crash in France, from July 2005 to December 2015, and identified by their national identifier were included. The association with crash risk was estimated using a responsibility analysis comparing the use of benzodiazepines and z-hypnotics among drivers responsible or not for the crash.

**Results:**

A total of 97,936 responsible and 103,522 non-responsible drivers involved in an injurious crash were included. The proportion of drivers exposed to benzodiazepine anxiolytics or z-hypnotics remained stable among responsible and non-responsible drivers. Among controls from the general population, the proportion of exposed individuals tended to increase. The association with crash risk remained almost constant over the study period. The odds-ratio for benzodiazepines ranged between 1.42 [1.24–1.62] at the beginning of the study period and 1.27 [1.09–1.47] at the end.

**Conclusion:**

Given the increase in exposure in the control group from the general population, the stability of exposure for responsible and non-responsible drivers can be interpreted as a relative effectiveness of the pictogram on driver exposure levels. On the other hand, while the intrinsic effect of a prescription drug cannot be modified, a decrease in risk could have been expected if drivers adapted their behavior as promoted by the pictogram. Our results therefore suggest that no significant change occurred in driving behaviors or consumption patterns.

## Background

**Fig. 1:**
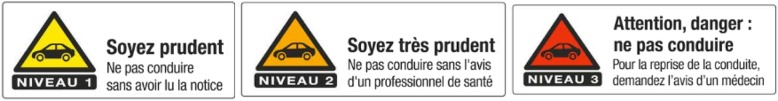
Three-level pictogram. *Level 1: Be careful. Read carefully the patient leaflet before driving. Level 2: Be very careful. Take advice from a physician or a pharmacist before driving. Level 3: Danger: do not drive. Seek medical advice before driving again*

Awareness of the involvement of prescription drugs as a risk factor for road traffic crashes dates back twenty years. The authorities in charge of road safety in France have decided to warn visually of the potentially harmful effects of prescription drugs on driving performance. Thus, in 1999, a unique triangular pictogram was designed to be affixed to the outer packaging of prescription drugs (Arrêté du 3 mai 1999 pris pour l’application de l’article R. 5143 du code de la santé publique et relatif à l’apposition d’un pictogramme Sur le conditionnement de certains médicaments (decree of may 3, 1999 for the implementation of article R. 5143 of the code of public Health on the adoption of a pictogram on the packaging of certain drugs and products), n.d.). This pictogram was printed on almost one-third of the drug boxes, some of which presented very high risks to driving while others only required a warning. This system did not allow this important distinction to be made. In response to these limitations, the French National Agency for Medicines and Health Products Safety decided in 2003, to implement a standardized classification of prescription drugs according to four levels of impaired driving, from level 0 (no or negligible risk) to level 3 (major risk) with three corresponding pictograms for the levels 1, 2 and 3 (Fig. 1), replacing the unique pictogram (Arrêté du 8 août 2008 pris pour l'application de l'article R. 5121-139 du code de la santé publique et relatif à l'apposition d'un pictogramme sur le conditionnement extérieur de certains médicaments et produits. (Decree of August 8, 2008 for the implementation of the article R. 5121-139 of the Code of Public Health on the adoption of a pictogram on the packaging of certain drugs and products), n.d.; Arrêté du 18 juillet 2005 pris pour l'application de l'article R. 5121–139 du code de la santé publique et relatif à l'apposition d'un pictogramme sur le conditionnement extérieur de certains médicaments et produits. (Decree of July 1 8, 2005 for the implementation of the article R. 5121–139 of the Code of Public Health on the adoption of a pictogram on the packaging of certain drugs and products), n.d.). In the absence of epidemiological data, our laboratory set up an observatory in 2005 to monitor road crash risks related to the use of prescription drugs in France in order to assess the relevance of the classification and the efficiency of the warning system. The linkage of police data on crashes with those from the health insurance system has made it possible to study the risks associated with the consumption of all pharmaceutical drugs (Orriols et al., 2010). Due to their high levels of consumption and association with crash risk, the two classes most represented in terms of attributable fraction were benzodiazepine anxiolytics and z-hypnotics (zolpidem and zopiclone) (Barbone et al., 1998; Chang et al., 2013; Engeland et al., 2007; Gjerde et al., 2015; Gustavsen et al., 2008; Hemmelgarn et al., 1997; Orriols et al., 2011; Ravera et al., 2011; Smink et al., 2010). A first observation of exposure trends to benzodiazepine anxiolytics and z-hypnotics among drivers involved in road traffic crashes was made, suggesting a transient downward trend in exposure following the implementation of the color-graded pictogram system (Orriols et al., 2016). Given that consumption levels of psychotropic prescription drugs are likely to change rapidly, particularly among drivers whose average age is increasing, it seems necessary to continue to monitor the level of exposure to prescription drugs and the resulting risk of road traffic crashes.

The objective of this study was to describe the evolution of exposure to benzodiazepine anxiolytics and z-hypnotics and the association between exposure to these prescription drugs and responsibility for a road traffic crash over the period 2005–2015 in France.

## Methods

### Data sources

The study was based on three French nationwide databases: police reports (PR), the national police database of injurious crashes (IC) and the national healthcare insurance database (HCI database) (Orriols et al., 2010).

#### Police reports (PRs)

The French police forces are required to complete a report for each injurious crash occurring in the country. They are scanned and stored as image files. For some of the individuals involved, the national ID number (NID, also called social security number) is recorded in the PR. A manual evaluation on a small sample of reports estimated that the NID was recorded for 28% of the drivers involved (11). These NIDs were extracted from PR image files for subsequent linkage with dispensing records from the HCI database.

#### National police database of injurious road traffic crashes (ICs)

Police forces also collect details about each injurious crash, which are stored in the IC database (Bulletins d’Analyse des Accidents Corporels [BAAC]). This database contains all information on the characteristics of the crash, the vehicles and the people involved in the crash. Police forces also conduct additional investigations into the severity of injuries from hospital records and classify those involved into four groups: unhurt, slightly injured, seriously injured (hospitalized for more than 24 h), or killed (within 30 days of the crash). All drivers involved in an injurious crash must be tested for the presence of alcohol, using a breathalyzer. If this test is positive (≥0.5 g/l), the driver refuses the test, or the severity of the crash makes it impossible to administer the test, the blood alcohol level is measured. If the breath test is negative, the driver is recorded as not under the influence of alcohol.

#### National health-care insurance database (HCI database)

The HCI database (Système National d’Informations Inter Régimes de l’Assurance Maladie [SNIIR-AM]) covers the entire French population. A record is added each time a reimbursed prescription drug is dispensed to an outpatient in a pharmacy, including the NID, the date of dispensing and the seven-digit code that identifies the prescription drugs. In France, patients are fully reimbursed for health care expenses related to 30 recognized long-term chronic diseases (Jauregui et al., 2006), so these diseases are recorded in the HCI database with their ICD-10 code (International Classification of Diseases, Tenth Revision) and the start and end dates of the disease.

### Extraction and linkage procedures

The first step of the study was to extract the NID of drivers from the PRs by an automatic procedure. The NID number was used to link drivers to prescription drug reimbursement data around the date of the crash. The PRs were linked to the IC database records by a probabilistic linkage method (11).

### Participant inclusion

The drivers included in the study are those involved in a road crash in France from 2005 to 2015 whose NID, gender and date of birth were correctly entered in the PRs. They were excluded when the PR data extraction procedure failed or when the link between the PR database and the IC database could not be established. If a driver was involved in more than one crash during the study period, only the first crash was considered to ensure that prescription drug dispensations were not the result of a previous crash.

### Determining drivers crash responsibility

Drivers responsibility in the crash was determined using a standardized method adapted from Robertson and Drummer (Robertson & Drummer, 1994). The principle of responsibility analysis is to compare the probabilities of exposure on the day of the crash between the drivers deemed responsible for the crash and those non-responsible. This method ensures that both responsible and non-responsible drivers are selected from the driver population. This method, which has already been validated in France using data from the national police database of fatal crashes (Laumon et al., 2005), takes into account the various factors that may reduce driver responsibility: road, vehicle and driving conditions, type of crash, compliance with traffic rule and the difficulty of the task. A score is assigned to each driver for each of these factors, from 1 (favorable for driving) to 4 (not favorable for driving). The higher the sum of the scores, the less favorable the driving conditions are and, consequently, the more the driver will be considered not responsible for the crash. Drivers were then grouped into two levels of responsibility for the crash: responsible (score < 15) or non-responsible (score ≥ 15).

### Control group from the general population

To estimate the frequency of exposure to benzodiazepine anxiolytics and z-hypnotics, a control group was randomly selected from the entire national HCI database. The control sample included people from the general population who had not been selected to be involved in a road traffic crash. However, it is possible that a very small proportion of them were involved in a crash during the study period. The controls were individually matched to drivers (responsible and non-responsible) by gender and age. Prescription drug exposure was estimated based on the crash date of the drivers with whom the control was matched. This sample was only available from 2008 onwards because data from previous periods had already been archived.

### Prescription drugs and exposure periods

Daily exposure to prescription drugs has been estimated for benzodiazepine anxiolytics and z-hypnotics (zolpidem and zopiclone) and other prescription drugs classified as risk levels 2 or 3 for impaired driving. Exposure to prescription drugs was considered to begin on the day following delivery. The duration of exposure was estimated from the median values reported in a survey on drug prescription (EPPM) in France (Health, 2005). This survey was conducted among 800 practitioners, representative of French physicians, three times a year, over a 7-day period. To ensure that the prescribed drugs were not a consequence of the crash, the prescription drugs delivered on the day of the crash were not included in the analysis.

### Time periods

The study period was divided into six periods. The first (July 2005–December 2006) was a period during which the three-level pictogram had not yet been implemented. Indeed, in 2005, the list of drugs and corresponding pictograms were published as an official regulation and pharmaceutical companies had to comply with it during the following year. The second period (January 2007–May 2008) was set immediately after the introduction of the three-level pictogram. Four others periods were defined in order to have groups of equivalent size throughout the study: June 2008–December 2009 (period 3), January 2010–December 2011 (period 4), January 2012–October 2013 (period 5), and November 2013–December 2015 (period 6).

### Statistical analysis

The data were analyzed using the RStudio software, version 1.0.153 –© 2009–2017 RStudio, Inc.

#### Descriptive analysis

Driver characteristics were described over the six time periods. The frequency of exposure to benzodiazepine anxiolytics and z-hypnotics was calculated for the three groups (responsible drivers, non-responsible drivers, and controls from the general population) for all six periods. Exposure percentages were standardized on age groups in the first study period using a direct standardization method. Trends over the study period were tested using the Cochrane Armitage test.

#### Univariate and multivariate analysis

The association between driver responsibility in the crash and exposure to benzodiazepine anxiolytics and z-hypnotics was investigated by univariate and multivariate logistic regression complete case analysis for each of the six periods. Complete cases were defined as cases with no missing values for any of the variables.

The adjustment variables were included in multivariate model based on factors identified in the literature as potential risk factors or confounding factors:

- socio-economic characteristics: age, gender and socio-economic category.
- health characteristics: other prescription drugs of risk level 2 or 3 for impaired driving (including antiepileptics, psycholeptics, psychoanaleptics, opioids and some antihistamines), and long-term chronic diseases (including diabetes, severe neuromuscular disorders and long-term psychiatric disorders).
- alcohol level
- characteristics of the crash: type of vehicle, month, day of the week and time of day, location of the crash, severity of injuries;

We tested the interaction between benzodiazepine anxiolytics and z-hypnotics and age, gender, exposure to other prescription drugs of risk level 2 or 3 for impaired driving, alcohol, study period and long-term chronic diseases. Data were missing only for blood alcohol level (15%). The assumption of missing data at random was considered plausible. Therefore, the sensitivity analysis performed was based on multiple imputation by chained equations according to the Rubin rule, using MICE package to manage missing data (Robin, 1987; Van Buuren & Groothuis-Oudshoorn, 2011). According to the literature, we imputed fifteen databases corresponding to percentages of missing data in ten cycles. The variables to be explained and all covariates to be included in the multivariate analysis were included in the imputation model. The sensitivity analysis consisted of a multivariate analysis from imputed databases.

### Ethics statement

Confidentiality was ensured by using the anonymization function of the HCI system (14). The study was approved by the French data protection authority (Commission Nationale Informatique et Libertés).

## Results

**Fig. 2:**
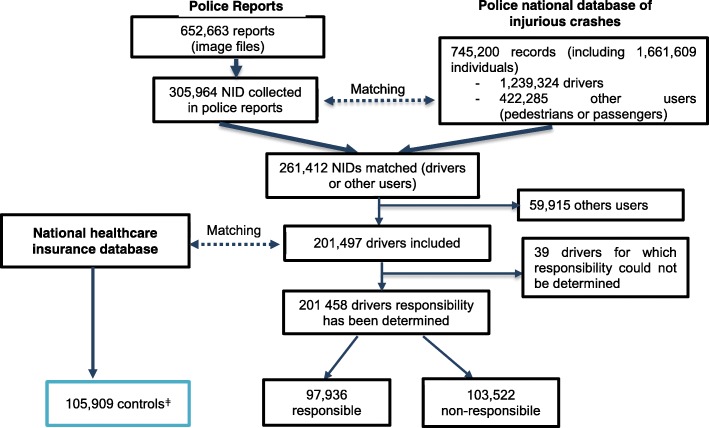
Flowchart of the inclusion procedure. *ǂ The control group was available from 2008 to 2015 only*

We extracted 305,964 NID/gender/date of birth from a total of 652,663 police reports available during the study period. Approximately 85% of these individual were linked to the corresponding record in the IC database. The discrepancy between the number of records in the IC and in the PR databases is explained by the fact that a small proportion of the reports were used for ongoing legal investigations and were therefore unavailable. 59,915 NIDs were excluded because they were other type of road users (pedestrians, passengers...). After extraction, linkage procedures and exclusion of other type of road users, 201,497 drivers were included (16.3% of drivers in the IC database). Responsibility in the crash was established for 201,458 drivers: 97,936 were identified as responsible and 103,522 as not responsible (Fig. 2). The control group from the HCI database was not requested at the beginning of the study and was therefore only available for the period 2008–2015, including 105,909 subjects.

The proportion of men was significantly higher among responsible than among non-responsible drivers. Drivers under 24 and over 65 years of age were more represented among responsible than among non-responsible drivers (Table 1). The controls were individually matched to drivers (responsible and non-responsible) by gender and age. The socioeconomic category was not available for controls.

**Table 1:** Sample characteristics of drivers and responsibility for road traffic crash

Variables	Responsible		Non responsible	
	n	%	n	%
Total	97,936		103,522	
Age *				
≤ 24 years	25,421	26.0	19,651	19.0
25–44 years	41,179	42.0	47,060	45.5
45–64 years	23,008	23.5	30,055	29.0
≥ 65 years	8328	8.5	6756	6.5
Gender *				
Men	70,004	71.5	70,039	67.7
Women	27,932	28.5	33,483	32.3
Socioeconomic category *				
Professional driver	3178	3.2	3773	3.6
Farmer	583	0.6	656	0.6
Craftsman, shopkeeper, independant profession	3848	3.9	4208	4.1
High managerial and professional occupation	3984	4.1	5342	5.2
Middle manager, employee	23,787	24.3	32,156	31.1
Worker	16,269	16.6	13,853	13.4
Retired	10,206	10.4	9286	9.0
Unemployed	5265	5.4	3597	3.5
Other/missing	21,719	22.2	23,208	22.4
Student	9097	9.3	7443	7.2

* *p* <  0.001 (Chi-squared test)

**Table 2:** Comparison of driver characteristics over the six periods

Variables	Period 1		Period 2		Period 3		Period 4		Period 5		Period 6	
	n	%	n	%	n	%	n	%	n	%	n	%
Tota	40,657		32,028		33,395		36,683		30,757		27,977	
Age *												
≤ 24 years	10,113	24.9	7756	24.2	7360	22.0	8008	21.8	6393	20.8	5452	19.5
25–44 years	17,921	44.1	14,233	44.4	15,186	45.5	16,093	43.9	13,112	42.6	11,708	41.9
45–64 years	9929	24.4	7857	24.5	8614	25.8	9856	26.9	8671	28.2	8147	29.1
≥ 65 years	2694	6.6	2182	6.8	2235	6.7	2726	7.4	2581	8.4	2670	9.5
Gender*												
Men	27,904	68.6	21,866	68.3	23,581	70.6	25,463	69.4	21,557	70.1	19,701	70.4
Women	12,753	31.4	10,162	31.7	9814	29.4	11,220	30.6	9200	29.9	8276	29.6
Socioeconomic category *												
Professional driver	1383	3.4	900	2.8	984	2.9	1134	3.1	1128	3.7	1422	5.1
Farmer	212	0.5	174	0.5	166	0.5	224	0.6	228	0.7	235	0.8
Craftsman, shopkeeper, independant profession	1290	3.2	1150	3.6	1380	4.1	1668	4.5	1364	4.4	1204	4.3
High managerial and professional occupation	1518	3.7	1266	4.0	1789	5.4	1801	4.9	1553	5.0	1401	5.0
Middle manager, employee	10,954	26.9	8921	27.9	9709	29.1	10,425	28.4	8555	27.8	7394	26.4
Worker	6463	15.9	5424	16.9	5011	15.0	5365	14.6	4261	13.9	3603	12.9
Retired	3437	8.5	3012	9.4	3077	9.2	3624	9.9	3195	10.4	3153	11.3
Unemployed	1808	4.4	1213	3.8	1476	4.4	1692	4.6	1387	4.5	1288	4.6
Other/missing	9215	22.7	6799	21.2	7402	22.2	8125	22.1	6967	22.7	6424	23.0
Student	4377	10.8	3169	9.9	2401	7.2	2625	7.2	2119	6.9	1853	6.6
Injury severity *												
Unhurt	10,365	25.5	8728	27.3	10,122	30.3	11,643	31.7	9625	31.3	8555	30.6
Killed	861	2.1	540	1.7	485	1.5	529	1.4	358	1.2	421	1.5
Seriously injured	14,856	36.5	11,008	34.4	9909	29.7	11,217	30.6	10,141	33.0	9675	34.6
Slightly injured	14,575	35.8	11,752	36.7	12,879	38.6	13,294	36.2	10,633	34.6	9326	33.3
Alcohol (g/l) *												
< 0.5	32,207	79.2	26,275	82.0	26,946	80.7	29,994	81.8	25,690	83.5	23,517	84.1
[0.5–0.8[	281	0.7	193	0.6	134	0.4	135	0.4	219	0.7	169	0.6
[0.8–1.2[	379	0.9	332	1.0	264	0.8	349	1.0	324	1.1	271	1.0
[1.2–2.0[	946	2.3	710	2.2	633	1.9	772	2.1	712	2.3	609	2.2
≥ 2.0	767	1.9	676	2.1	612	1.8	641	1.7	528	1.7	422	1.5
Missing	6077	15.0	3842	12.0	4806	14.4	4792	13.1	3284	10.7	2989	10.7
Day *												
Monday – Friday	30,236	74.4	23,649	73.8	25,192	75.4	27,593	75.2	23,110	75.1	20,899	74.7
Saturday	5826	14.3	4739	14.8	4614	13.8	5135	14.0	4165	13.5	3764	13.5
Sunday	4595	11.3	3640	11.4	3589	10.7	3955	10.8	3482	11.3	3314	11.8
Time of day *												
[5–10]	9084	22.3	7496	23.4	7918	23.7	8674	23.6	7397	24.0	7089	25.3
[11–13]	6352	15.6	5078	15.9	5208	15.6	5577	15.2	4783	15.6	4409	15.8
[14–19]	18,289	45.0	14,451	45.1	14,887	44.6	16,815	45.8	14,027	45.6	12,544	44.8
[20–22]	3962	9.7	2974	9.3	3220	9.6	3360	9.2	2720	8.8	2369	8.5
[23–1]	1879	4.6	1275	4.0	1420	4.3	1385	3.8	1122	3.6	944	3.4
[2–4]	1091	2.7	754	2.4	742	2.2	872	2.4	708	2.3	622	2.2
Vehicle type *												
Bicycle	2185	5.4	1682	5.3	1851	5.5	1876	5.1	1752	5.7	1632	5.8
Scooter	5842	14.4	4257	13.3	3692	11.1	3510	9.6	2564	8.3	1938	6.9
Motorbike	5670	13.9	4788	14.9	6606	19.8	6814	18.6	5651	18.4	5152	18.4
Light vehicle	24,132	59.4	18,660	58.3	18,431	55.2	21,182	57.7	17,717	57.6	16,312	58.3
Commercial vehicle	1185	2.9	1365	4.3	1598	4.8	1820	5.0	1695	5.5	1653	5.9
Heavy goods vehicle	793	2.0	549	1.7	560	1.7	677	1.8	648	2.1	610	2.2
Other	850	2.1	727	2.3	657	2.0	804	2.2	730	2.4	680	2.4
Crash location *												
Extra-urban	18,025	44.3	13,951	43.6	12,410	37.2	15,566	42.4	14,416	46.9	14,405	51.5
1–5000 inhabitants	4498	11.1	3254	10.2	2828	8.5	3505	9.6	3327	10.8	3110	11.1
5001–50,000 inhabitants	9854	24.2	8235	25.7	7073	21.2	7593	20.7	5602	18.2	4962	17.7
50,001–300,000 inhabitants	6757	16.6	5431	17.0	4806	14.4	4915	13.4	3365	10.9	3679	13.2
≥ 300,000 inhabitants	1523	3.7	1157	3.6	6278	18.8	5104	13.9	4047	13.2	1821	6.5
Responsible *												
Yes	19,580	48.2	15,316	47.8	16,182	48.5	18,275	49.8	15,198	49.4	13,385	47.8
Level 2 prescription drugsǂ*												
Yes	4655	11.4	3613	11.3	3816	11.4	4486	12.2	3465	11.3	3121	11.2
Level 3 prescription drugsǂ*												
Yes	1116	2.7	866	2.7	850	2.5	1024	2.8	1013	3.3	890	3.2
Long-term chronic diseases *												
Yes	3407	8.4	2826	8.8	3048	9.1	3656	10.0	3457	11.2	3278	11.7

*ǂ Risk level for driving impairment.*

* p < 0.0001 (Chi-squared test)

**Fig. 3:**
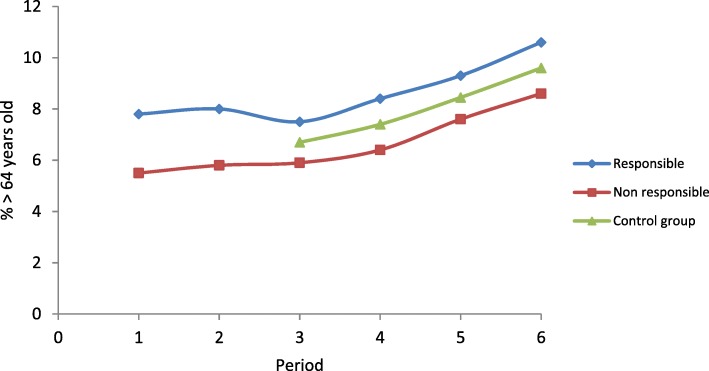
Percentage of subjects over 64 years of age among drivers (responsible and non-responsible) and controls by study periods

The distribution by gender was almost identical over the different periods. However, the proportion of subjects aged 65 years and over was higher in periods 4 to 6 (Fig. 3), which explains the increase during the same periods in the proportion of retired people and drivers with long-term chronic diseases on the day of the crash. The percentage of seriously injured drivers increased in periods 5 and 6 compared to period 4, while the percentage of slightly injured drivers decreased. The distribution of responsibility in the crash and exposure to level 2 and level 3 prescription drugs were very similar over the different time periods (Table 2).

**Fig. 4:**
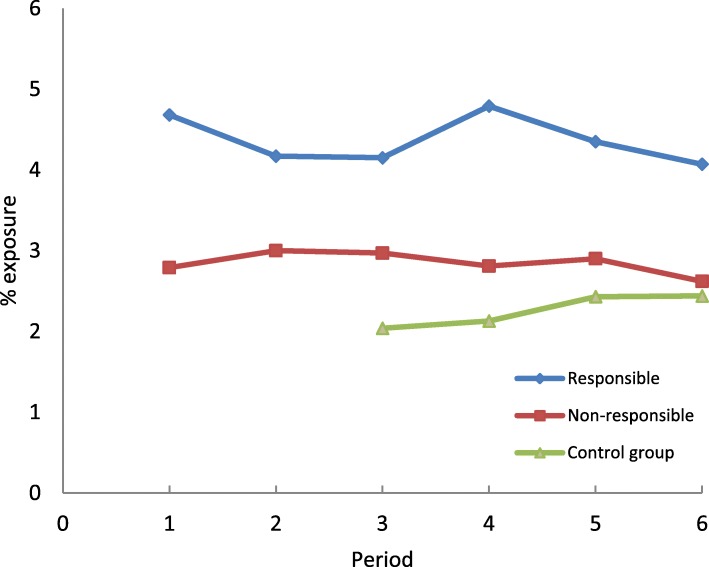
Frequency of exposure to benzodiazepine anxiolytics on the day of the crash in drivers (responsible and non-responsible) and controls according to study periods

The proportion of drivers exposed to benzodiazepine anxiolytics has remained stable over time among drivers (responsible or not responsible). Among controls from the general population, the proportion of exposed individuals increased significantly from period 3 to period 6 (Fig. 4). It should be noted that in order to take into account variations in the age structure, the figures have been standardized by taking the structure of the first period as a reference.

**Fig. 5:**
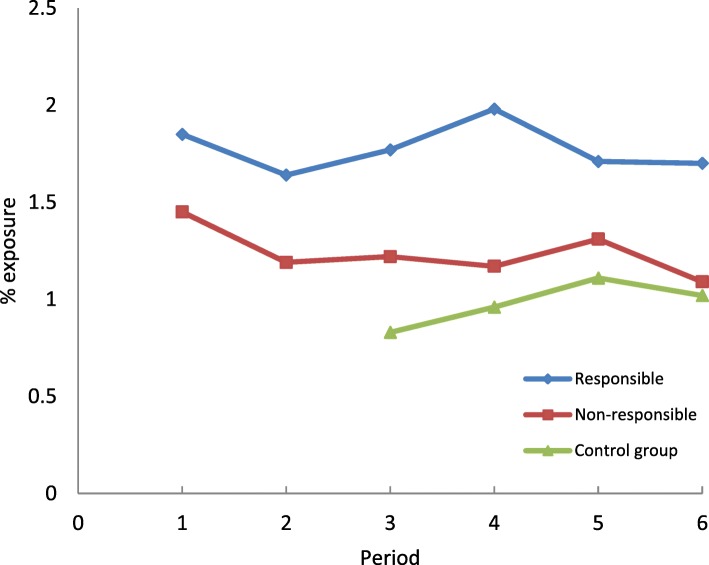
Frequency of exposure to z-hypnotics on the day of the crash in drivers (responsible and non-responsible) and controls according to study periods

The trends were similar for the exposure to z-hypnotics (Fig. 5).

Over all time periods, the frequency of exposure to benzodiazepine anxiolytics and z-hypnotics was higher among drivers responsible for the crash than among non-responsible drivers and controls.

After adjusting for the other variables, exposure to benzodiazepine anxiolytics was associated with driver responsibility in the crash at all times except for period 2 which corresponds to the introduction of the three-level pictogram. The odds-ratio ranged between 1.42 [1.24–1.62] in period 1 and 1.27 [1.09–1.47] in period 6. Exposure to z-hypnotics was also associated with responsibility with lower exposure levels and therefore a less consistent pattern in statistical significance (Table 3).

**Table 3:** Association between exposure to benzodiazepine anxiolytics and z-hypnotics and drivers responsibility in each study period

	Period 1 OR [95% CI]	Period 2 OR [95% CI]	Period 3 OR [95% CI]	Period 4 OR [95% CI]	Period 5 OR [95% CI]	Period 6 OR [95% CI]
benzodiazepine anxiolytics^ǂ^	1.42 [1.24–1.62]	1.11 [0.96–1.29]	1.23 [1.06–141]	1.38 [1.21–1.57]	1.18 [1.03–1.37]	1.27 [1.09–1.47]
z-hypnotics^ǂ^	1.01 [0.84–1.21]	1.23 [1.00–1.51]	1.38 [1.13–1.69]	1.37 [1.13–1.65]	1.04 [0.85–1.27]	1.38 [1.11–1.71]

ǂ reference = drivers not exposed; OR: Odds Ratio; CI: confidence interval.

Complete cases: Period 1: *N* = 34,580; Period 2: *N* = 28,186; Period 3: N = 28,589; Period 4: *N* = 31,891; Period 5: *N* = 27,473; Period 6: *N* = 24,988.

OR adjusted for age, gender, long-term chronic diseases, socioeconomic category, other prescription drugs of risk level 2 for driving impairment, other prescription drugs of risk level 3 for driving impairment, alcohol level, time of day, month, crash location, vehicle type.

No interactions were observed between exposure to benzodiazepine anxiolytics / z-hypnotics and age, gender, long-term chronic diseases, alcohol, exposure to other prescription drugs of risk level 2 or 3 for impaired driving and the study period. Sensitivity analyses showed that the estimates obtained after multiple imputation were identical to those obtained from the complete case analysis.

## Discussion

This study assessed the evolution of exposure to benzodiazepine anxiolytics and z-hypnotics and of the association with driver responsibility in road traffic crashes between 2005 and 2015 in France. The results showed stable exposure among drivers involved in road traffic crashes during the study period and a significant upward trend in controls from the general population not involved in crashes. The association with crash risk has remained almost constant.

Drivers exposed to benzodiazepine anxiolytics in period 1 had a higher risk of being responsible for the crash than unexposed drivers. This association disappeared in period 2, which corresponded to the introduction of three-level pictogram, but became significant thereafter. The decrease in exposure among responsible drivers observed in period 2 could be explained by the information provided to patients by healthcare professionals when the colour-graded pictogram was introduced (ANSM. Médicaments et conduite automobile. (Medicinal products and automobile driving, n.d.). The association between exposure to benzodiazepine anxiolytics and driver responsibility observed from period 3 to period 6 suggests that the impact of the pictogram on risk was not sustainable. An increased risk of being responsible for a crash was observed among drivers exposed to z-hypnotics in four of the six study periods considered. In controls from the general population, exposure to benzodiazepine anxiolytics and z-hypnotics increased over the study period. Since drivers aged 65 and over were more represented in periods 4 to 6 and benzodiazepine use increases with age, exposure prevalence estimates have been standardized by age group in the first period. The control group was matched to drivers by age and gender, so the age distribution was identical in these groups. Given the increase in exposure in the control group from the general population, the stability of exposure for responsible and non-responsible drivers could therefore be interpreted as a relative effectiveness of the pictogram on driver exposure levels. Indeed, if the pictogram had a significant impact, one would expect a decrease in driver exposure levels. On the other hand, we did not observe any decrease in the strength of the association between exposure and responsibility in the crash. While the intrinsic effect of a prescription drug cannot be modified, a decrease in risk would be expected if drivers adapted their behavior. In this study, drivers had unchanged risk, suggesting that there was no change in driving behaviors or consumption patterns.

To our knowledge, only one study assessed the effectiveness of pictograms in communicating road safety risks. This study interviews participants visiting community pharmacies in The Netherlands, with structured questions related to intention to change driving behavior when exposed to different pictogram models on prescription drug boxes, including the French model. The results showed that respondents’ intentions to change their driving behaviors increased with higher categories of risk. The authors also point out that the French labelling system, which has no frame of reference related to other levels of risk, can lead to underestimation of the hazards of drugs with the highest risk levels (Monteiro et al., 2013). This study reflects only the intention to change driving behaviors and not the actual behavior that would be adopted in a real situation.

In France, the use of benzodiazepine anxiolytics and z-hypnotics decreased between 2012 and 2015 (ANSM, 2017). In contrast, in our control group, the use of benzodiazepines and z-hypnotics increased significantly over the study period. The controls being matched by age and gender of drivers involved in road traffic crashes, they are not representative of the whole French population. Indeed, in our study, individuals aged more than 65 years old represented 9.5% of our sample in the last period while they were 18.6% in 2015 in France. In this age group, we also found a decrease in exposure between 2013 and 2015 (data not shown). Individuals in this age group are under-represented in our study, explaining the absence of a decrease in the last period of our study. Nevertheless, this matching procedure made it possible to compare exposures with responsible and non-responsible drivers. It should be noted, however, that we had no information on the driving status of controls.

The study has two main limitations. First, despite the inclusion of 201,497 drivers involved in a road traffic crash, giving our study unprecedented statistical power, the included drivers represented about 16% of all drivers in the national injury crash database over the study period. This is mainly due to the unsystematic presence of the NIDs in police reports. A previous study conducted in the same database compared included and non-included drivers and showed that severity of injuries was associated with the probability of being part of the study (Orriols et al., 2010). This may be explained by the fact that injured drivers were more likely to be admitted to hospital, so their healthcare number was more often mentioned in the police report. As a result, our sample slightly over-represented injured drivers in more severe crashes. The second limitation concerned exposure estimation. Indeed, exposure to prescription drugs was determined from computerized records of reimbursed prescriptions filled at the pharmacy. As a result, we had no information on compliance or self-medication. However, these data were not subject to underreporting, a major problem when prescription drug exposure data are self-reported. It should also be noted that we had no reliable information on illicit drug use and other behavioral factors that can lead to crashes (inattention, phone use while driving, etc.).

Responsibility analysis is a real strength of the study because it uses non-responsible drivers, who share common characteristics with responsible drivers, as a control group. In a previous study on the impact of illicit drug use, using the same police national database but limited to fatal crashes*,* the method used to determine responsibility was validated against an independent expert assessment of responsibility (Laumon et al., 2005). It is important to note that responsibility levels have been calculated independently of alcohol and illicit drug use because of their potential interactions with prescription drugs use. It should be also noted that the inability of non-responsible drivers to avoid a crash could be also related to prescription drugs, a risk that could not be taken into account in the design of our study. This scenario is plausible since the level of exposure in the control group was generally lower than that of drivers who were not responsible.

## Conclusion

The previous analysis of 2005–2011 data suggested an increase in exposure to benzodiazepine anxiolytics and z-hypnotics in 2010–2011 (Orriols et al., 2016). The follow-up conducted as part of this study in subsequent years showed that the use of these prescription drugs among drivers involved in a road traffic crash is stabilizing. The association with crash risk remained constant over the study period, despite the implementation of the three-level pictogram system. However, the pictogram probably had an impact on exposure levels to benzodiazepine anxiolytics and z-hypnotics.
